# Supplementary material for: The nuclear receptor NR4A1 is regulated by SUMO modification to induce autophagic cell death
Source: PLoS One. 2020 Mar 25;15(3):e0222072. doi: 10.1371/journal.pone.0222072 (PMC7094859; doi:10.1371/journal.pone.0222072)
Supplement: S1 Raw images — (PDF) [file pone.0222072.s001.pdf]

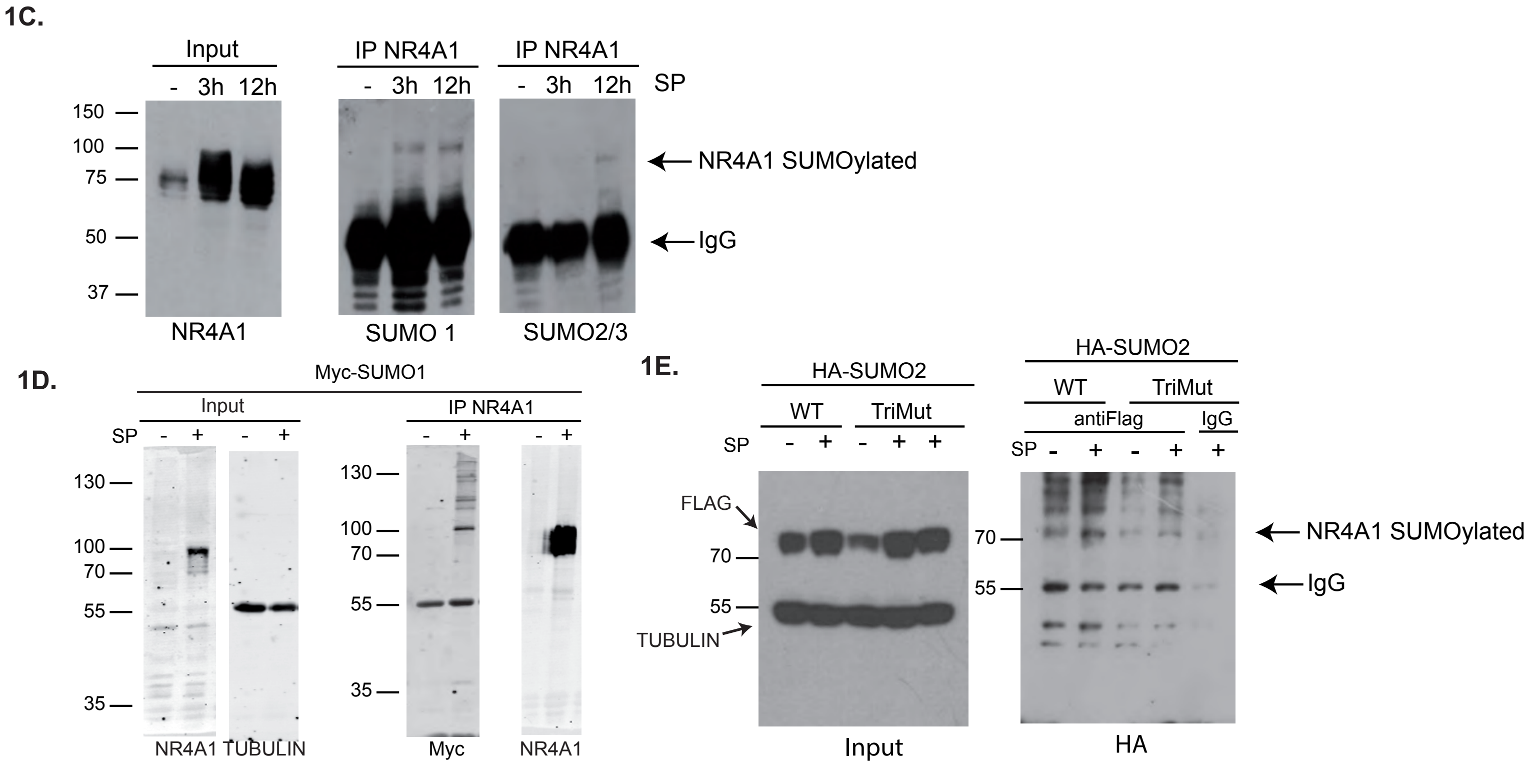

Blots corresponding to Figure 1.

These blots were developed by chemioluminiscence. Films were scanned.

2A.

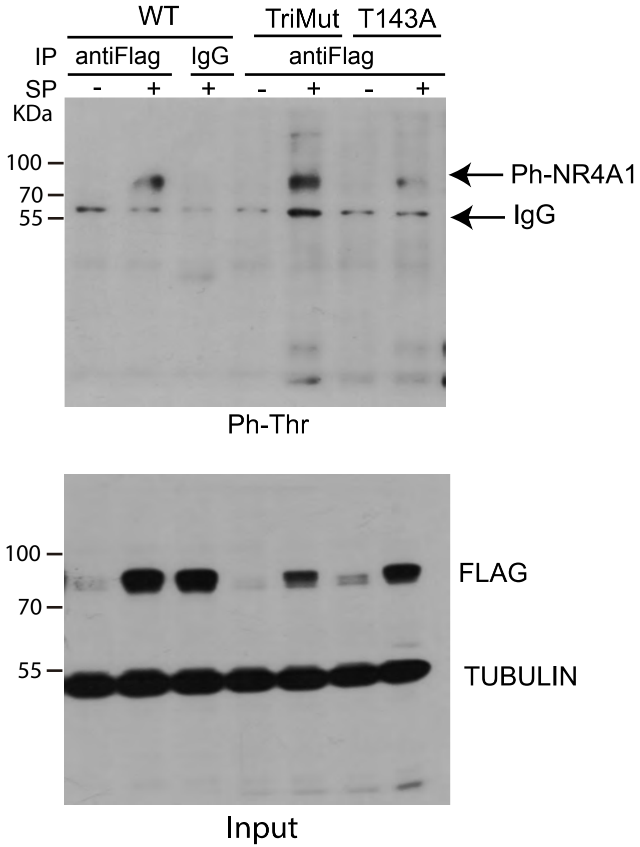

2B.

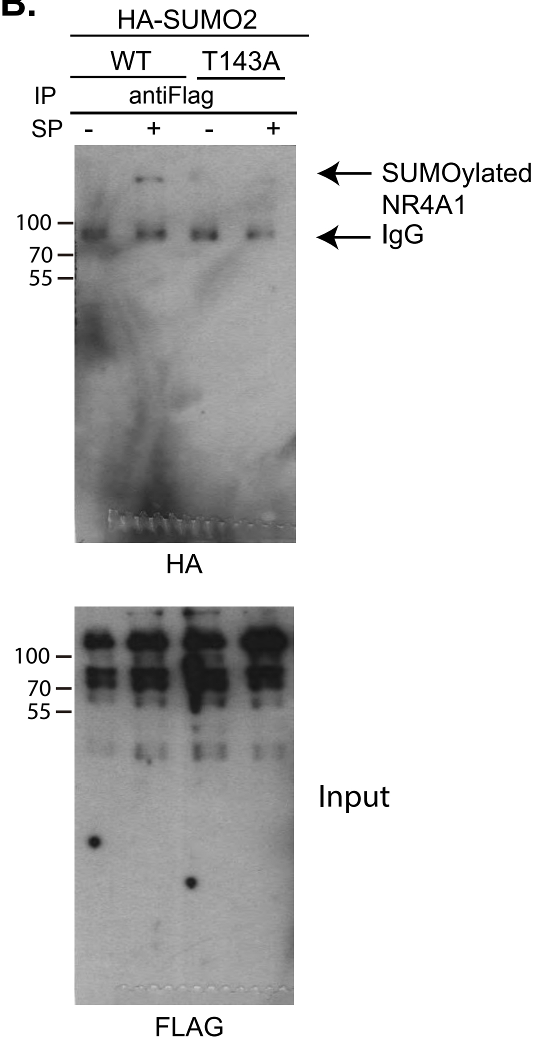

Blots corresponding to Figure 2A and 2B.

These blots were developed by chemioluminiscence. Films were scanned.

2C.

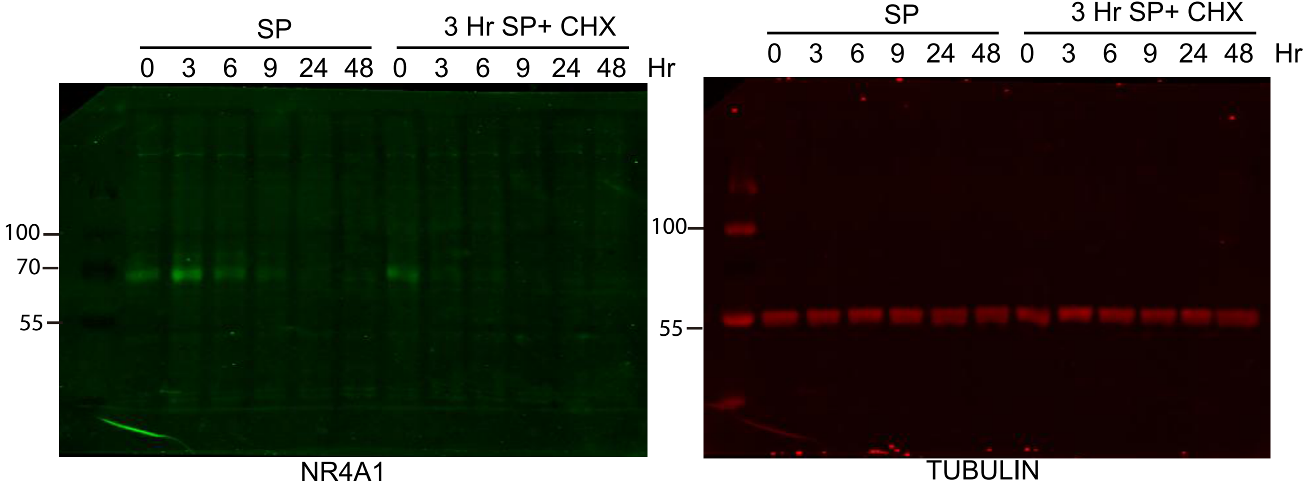

Blots corresponding to Figure 2C.
NR4A1 and TUBULIN were detected simultaneously by Western blot, developed with infrared dye-coupled secondary antibodies and scanned in an Odyssey Imager.

2D.

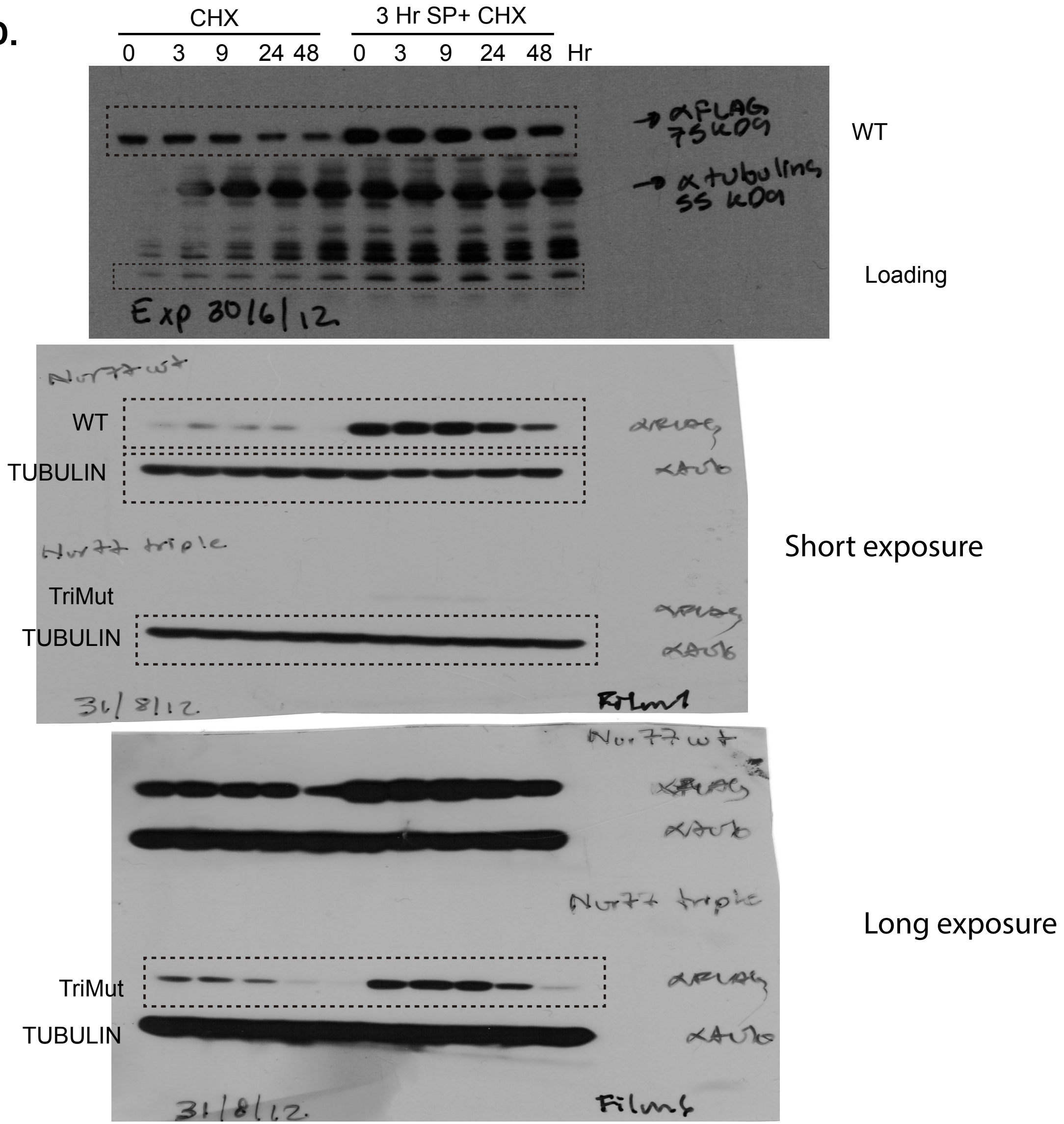

Figure 2D.
Blots corresponding to Figure 2D.
These blots were developed by chemioluminiscence. Films were scanned.

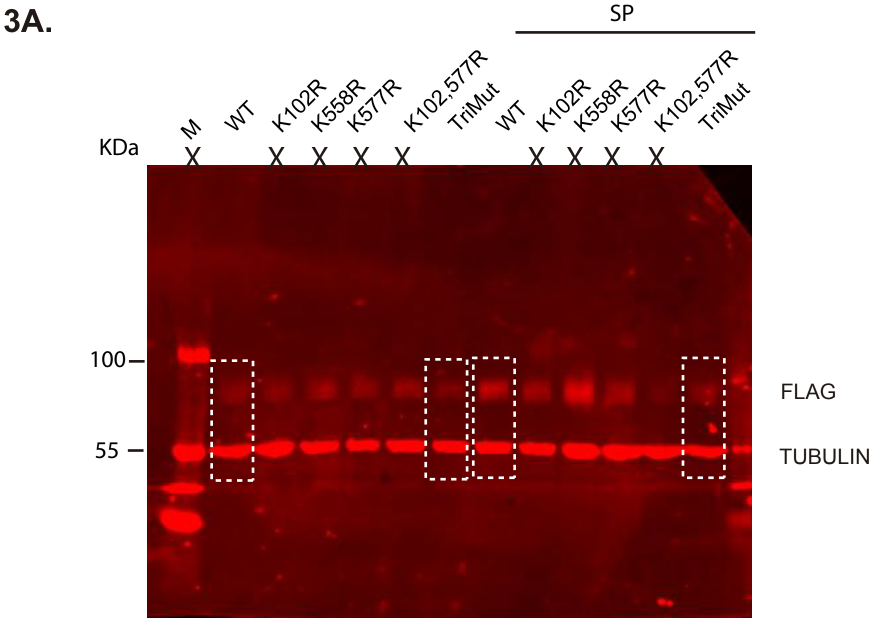

Blots corresponding to Figure 3A.

FLAG and TUBULIN were detected simultaneously by Western blot, developed with infrared dye-coupled secondary antibodies and scanned in an Odyssey Imager.

4A.

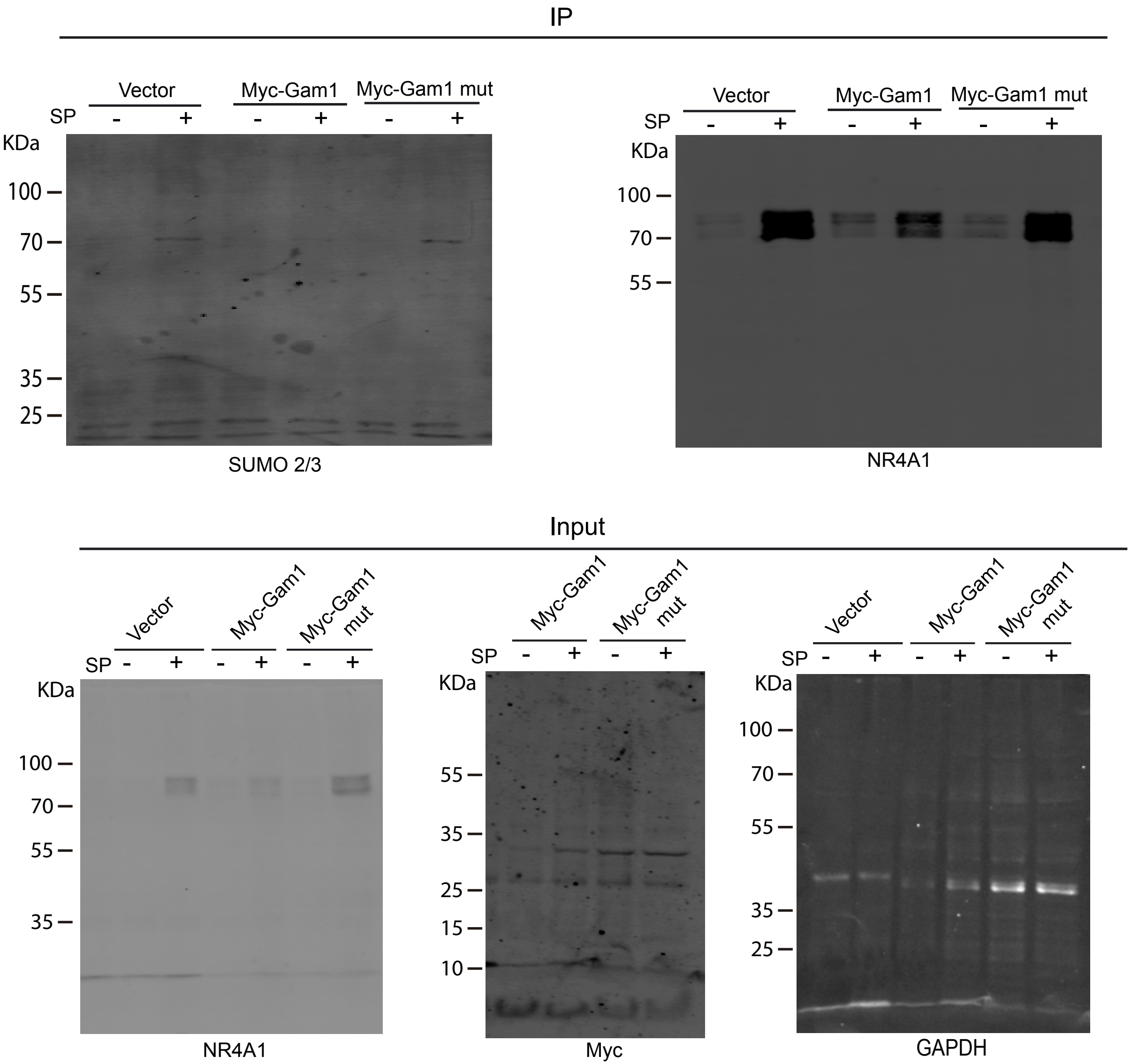

Blots corresponding to Figure 4A.

These blots were developed by chemioluminiscence. Films were scanned.

4B.

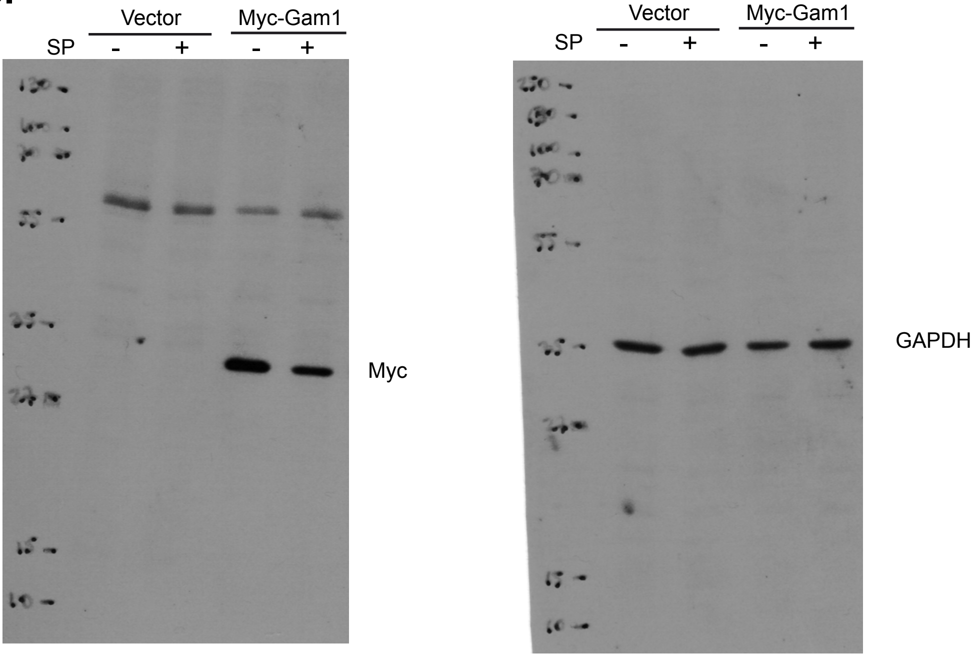

Blots corresponding to Figure 4B.
These blots were developed by chemioluminiscence. Films were scanned.

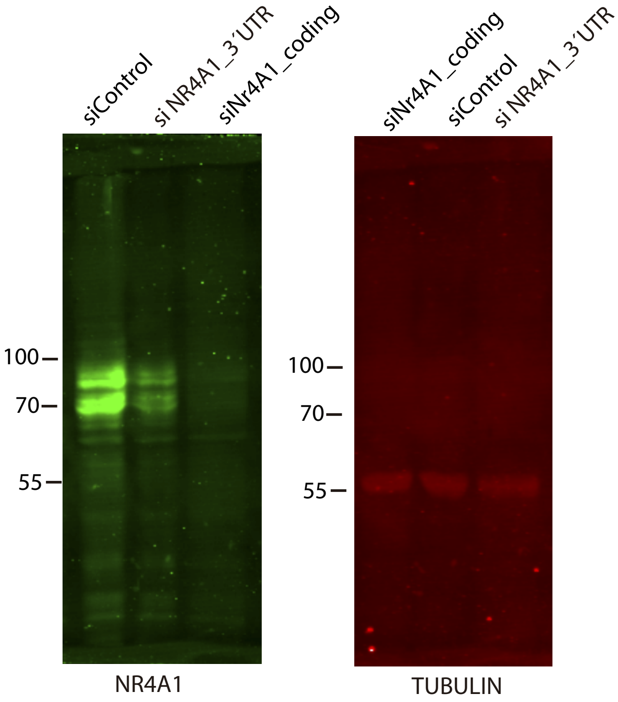

Methodology used in Fig. 4C: siRNA targeting *Nnr4a1* mRNA at the 3´UTR silences the expression of endogenous *Nr4a1,* allowing the expression of transfected *Nr4a1*. Cells were co-transfected with NK1R and NR4A1 WT expression vectors, as well as with a control siRNA targeting a viral sequence not present in mammals, a siRNA targeting the 3' UTR of *Nr4a1*, or a siRNA targeting the coding sequence of *Nr4a1*.Total protein extracts were obtained 3hr after SP addition to estimate the content of NR4A1 by WB. Tubulin was detected as a loading reference. The blots were developed using appropriate infrared dye-coupled secondary antibodies (1:10,000 dilution of anti-rabbit IRDye800 and anti-mouse IRDye700, Rockland, Gilbertsville, PA, USA) and scanned in an Odyssey Imager. Notice that the level of NR4A1 is partially reduced with siNR4A1_3'UTR, since the expression of endogenous *Nr4a1* was prevented. The expression of transfected *Nr4a1* expression vector is not affected, as this construct lacks the 3'UTR sequence. When a siRNA targeting the coding region was used, both endogenous and exogenous *Nr4a1* expression was reduced.
The images acquired for the blots shown in figure 4C were cropped and then saved. There were no extra bands in the blots obtained with these antibodies and develop method, as demonstrated in the whole blots shown in this figure.
